# Assessment of branch point prediction tools to predict physiological branch points and their alteration by variants

**DOI:** 10.1186/s12864-020-6484-5

**Published:** 2020-01-28

**Authors:** Raphaël Leman, Hélène Tubeuf, Sabine Raad, Isabelle Tournier, Céline Derambure, Raphaël Lanos, Pascaline Gaildrat, Gaia Castelain, Julie Hauchard, Audrey Killian, Stéphanie Baert-Desurmont, Angelina Legros, Nicolas Goardon, Céline Quesnelle, Agathe Ricou, Laurent Castera, Dominique Vaur, Gérald Le Gac, Chandran Ka, Yann Fichou, Françoise Bonnet-Dorion, Nicolas Sevenet, Marine Guillaud-Bataille, Nadia Boutry-Kryza, Inès Schultz, Virginie Caux-Moncoutier, Maria Rossing, Logan C. Walker, Amanda B. Spurdle, Claude Houdayer, Alexandra Martins, Sophie Krieger

**Affiliations:** 1 Laboratoire de Biologie Clinique et Oncologique, Centre François Baclesse, Caen, France; 2 Inserm U1245, Normandy Center for Genomic and Personalized Medicine, Rouen, UNIROUEN, Normandy University, Caen, France; 3 Université Caen-Normandie, Caen, France; 4 Interactive Biosoftware, Rouen, France; 5 Inserm UMR1078, Genetics, Functional Genomics and Biotechnology, Université de Bretagne Occidentale, Brest, France; 6 Inserm U916, Département de Pathologie, Laboratoire de Génétique Constitutionnelle, Institut Bergonié, Bordeaux, France; 7 Service de Génétique, Institut Gustave Roussy, Villejuif, France; 8 Lyon Neuroscience Research Center–CRNL, Inserm U1028, CNRS UMR 5292, University of Lyon, Lyon, France; 9 Laboratoire d’Oncogénétique, Centre Paul Strauss, Strasbourg, France; 10 Service de Génétique, Institut Curie, Paris, France; 11 Centre for Genomic Medicine, Rigshospitalet, University of Copenhagen, Copenhagen, Denmark; 12 Department of Pathology and Biomedical Science, University of Otago, Christchurch, New Zealand; 13 Department of Genetics and Computational Biology, QIMR Berghofer Medical Research Institute, Herston, Queensland Australia; 14 Present address: Laboratoire de biologie et génétique des cancers, Centre François Baclesse, Caen, France

**Keywords:** Branch point, Prediction, RNA, Benchmark, HSF, SVM-BPfinder, BPP, Branchpointer, LaBranchoR, RNABPS, Variants

## Abstract

**Background:**

Branch points (BPs) map within short motifs upstream of acceptor splice sites (3’ss) and are essential for splicing of pre-mature mRNA. Several BP-dedicated bioinformatics tools, including HSF, SVM-BPfinder, BPP, Branchpointer, LaBranchoR and RNABPS were developed during the last decade. Here, we evaluated their capability to detect the position of BPs, and also to predict the impact on splicing of variants occurring upstream of 3’ss.

**Results:**

We used a large set of constitutive and alternative human 3’ss collected from Ensembl (*n* = 264,787 3’ss) and from in-house RNAseq experiments (*n* = 51,986 3’ss). We also gathered an unprecedented collection of functional splicing data for 120 variants (62 unpublished) occurring in BP areas of disease-causing genes. Branchpointer showed the best performance to detect the relevant BPs upstream of constitutive and alternative 3’ss (99.48 and 65.84% accuracies, respectively). For variants occurring in a BP area, BPP emerged as having the best performance to predict effects on mRNA splicing, with an accuracy of 89.17%.

**Conclusions:**

Our investigations revealed that Branchpointer was optimal to detect BPs upstream of 3’ss, and that BPP was most relevant to predict splicing alteration due to variants in the BP area.

## Background

Pre-mRNA splicing by the spliceosome is essential for maturation of mRNA. Moreover, splicing plays a crucial role for protein diversity in eukaryotic cells [1]. This process, named alternative splicing, produces several mRNA molecules from a single pre-mRNA molecule and concerns approximately 95% of human genes [2]. RNA splicing requires a mandatory set of splicing signals including: the splice donor site (5’ss), the splice acceptor site (3’ss) and the branch point (BP) site. The 5’ss defines the exon/intron junction at the 5′ end of each intron with two highly conserved nucleotides, mainly GT. The 3’ss delineates the intron/exon junction at the 3′ end of each intron and is characterized by a highly conserved dinucleotide (mainly AG), which is preceded by a cytosine and thymidine rich sequence called the polypyrimidine tract. The branch site is a short motif upstream of the polypyrimidine tract that includes a BP adenosine, in 92% of human BP [3]. During the first step of the splicing reaction the 2’OH of the BP adenosine attacks the first intronic nucleotide (nt) of the upstream 5’ss to form a lariat intermediate [4]. In the second step, the 3’OH of the 5′ exon attacks the downstream 3’ss thereby releasing the intronic lariat and joining the two exons together.

The 5’ss and 3’ss sequences are well characterized, mostly having been experimentally mapped, which allowed the assembly of large datasets of aligned sequences [5–7]. Therefore, several reliable in silico tools dedicated to splice site predictions emerged, reaching an accuracy of 95.6% [8]. In contrast, the branch sites are short and degenerate motifs that are still poorly known and difficult to predict [3]. Indeed, only the branch A and the T located 2 nucleotides (nt) upstream, are highly conserved within a 5-mer motif of CTRAY [9]. More than 95% of BPs are located between 18 and 44 nt upstream of 3’ss [10], hereafter named the BP area. However, some BPs can be located up to 400 nt upstream of the 3’ss [11]. The identification of relevant BPs, i.e. BPs used by the spliceosome, represents a major challenge given the high variability of these BPs, both at localization and motif level. Disease-causing variants have most frequently been shown to be splicing motif alterations [12] and these variants can also alter BPs [13]. An accurate prediction of BP alteration represents a challenge to molecular diagnosis.

**Fig. 1:**
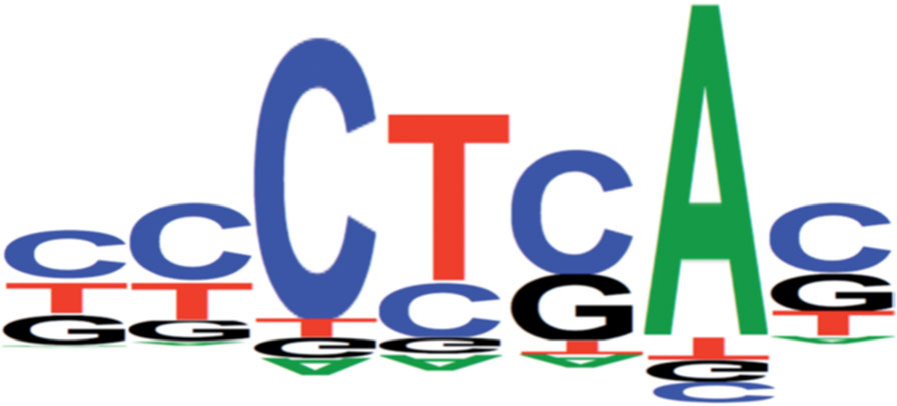
Illustration of position weight matrix used by HSF [14]

A major limit to develop accurate BP prediction tools was the limited access to experimentally-proven BPs. The first tools Human Splicing Finder (HSF) [14] and SVM-BPfinder [15] used only 14 and 35 experimentally-proven BPs in development. In 2015, a large but not comprehensive dataset of BPs was built from lariat RNA-seq experiments [10]. This collection of BPs was extended by two further studies: the first used 1.31 trillion reads from 17,164 RNA-seq data sets [16], and the second identified BPs by the spliceosome iCLIP method [17]. Thus, several bioinformatics tools for BP prediction have recently emerged: Branch Point Prediction (BPP) [18], Branchpointer [19], LaBranchoR [20] and RNA Branch Point Selection (RNABPS) [21] (Table 1). Briefly, HSF uses a position weighted matrix approach with a 7-mer motif as a reference (5 nt upstream and 1 nt downstream of the branch point A) (Fig. 1). SVM-BPfinder was the first to take into account, not only the branch site motif, but also the conservation of 3’ss, as well as the AG exclusion zone algorithm (AGEZ) [11] derived from the work of Smith and collaborators [23]. BPP combines the BP and 3’ss sequences and the AGEZ algorithm by a mixture model, a popular motif inference method. Branchpointer uses machine learning algorithms trained from a set of experimentally proven BPs. LaBranchoR and RNABPS are based on a deep-learning approach. LaBranchoR re-used the dataset of Branchpointer and implemented a bidirectional long short-term memory network (LSTM) that was shown to be performant for modeling sequential data such as natural language. RNABPS, as LaBranchoR, used the LSTM model and also implemented a dilated convolution neural network algorithm.

**Table 1:** Bioinformatics tools for branch point analyses, Human Splicing Finder (HSF), SVM-BPfinder, Branch Point Prediction (BPP), Branchpointer, LaBranchoR, RNA Branch Point Selection (RNABPS), with their main features and their accessibility

Tools	Features	Input	Accessibility	Refs
HSF	• Position weighted matrix of 7-mers (YNYCRAY)	DNA sequences^1^ or variants^1^ (nomenclature HGVS^2^)	Available as a web-application http://www.umd.be/HSF3/	[14]
	• Train on conserved sequences from the Ensembl transcripts			
SVM-BPfinder	• Support vector machine combining BP predictions and PPT^3^ features	DNA sequences (between 20 and 500 nt length)	Available as a web-application + Perl script http://regulatorygenomics.upf.edu/Software/SVM_BP/	[15]
	• Train on conserved sequences from 7 mammalian species (with Human)			
BPP	• Mixture model combining BP predictions and PPT^3^ features	DNA sequences (unlimited sequence length)	Available as a python script https://github.com/zhqingit/BPP	[18]
	• Train on conserved sequences from human introns			
Branchpointer	• Machine learning taking into account the primary and secondary structure of the RNA molecule	Text files with genomic coordinates (format defined by Branchpointer)	Available as an R Bioconductor package https://www.bioconductor.org/packages/release/bioc/html/branchpointer.html	[19]
	• Train on high-confidence BPs [10]			
LaBranchoR	• Deep learning based on bidirectional LSTM^4^ network	DNA sequences (70 nt upstream of the di-nucleotide AG)	Available as a python script + UCSC genome browser http://bejerano.stanford.edu/labranchor/	[20]
	• Train on high-confidence BPs [10]			
RNABPS	• Deep learning based on dilated convolution and bidirectional LSTM^4^ network	DNA sequences (70 nt upstream of the di-nucleotide AG)	Available as a web-application https://home.jbnu.ac.kr/NSCL/rnabps.htm	[21]
	• Train on high-confidence BPs [10] plus [16]			

^1^ Batch analyses are not available; ^2^ *HGVS* Human Genome Variation Society [22], https://varnomen.hgvs.org/; ^3^ *PPT* PolyPyrimidine Tract; ^4^ *LSTM* Long Short-Term Memory

Here, we present a benchmarking of these six BP-dedicated bioinformatics tools on their capacity to detect a relevant BP signal and to predict a variant-induced BP alteration. The resolution of the first issue allowed highlighting the specificity of each tool, i.e. the identification of BPs among background noise. For this part, we used two sets of data: a large set of 3’ss described in Ensembl database and a series of alternative 3’ss observed in RNA-seq experiments. The detection of BP alteration by a variant represents also a challenge for molecular diagnostics. To this end, we used an unprecedented collection of human variants (within the BP area) with their in vitro RNA studies to assess the prediction of variant effect on BP function.

## Results

### Bioinformatic detection of branch points among the physiological and alternative splice acceptor sites

In this study, two sets of 3’ss data were used, 3’ss described in Ensembl dataset and alternative 3’ss with their expression data from RNA-seq analyses (Table 2). The running times showed that BPP is one of the faster tools and Branchpointer one of the slower tools (Additional file 1: Figure S3).

**Table 2:** Summary of datasets used to compare the prediction tools

Name	Used	Origin	Control data	N (Positive / Control; %)
Ensembl data	Identification of BPs among background noise	3’ss supported by the transcripts described in Ensembl database	Any AG dinucleotides in the gene sequence	114,868,082 (264,787 / 114,603,295; 0.23%)
RNA-seq data	Correlation between expression of 3’ss and BP predictions	Alternative 3’ss observed in RNA-seq experiments	Random selection of 3’ss with MES score > 0	103,972 (51,986 / 51,986; 50%)
Variants collection	Detection of BP alteration by a variant	Variants occurring in the BP area (−44; −18) with in vitro RNA studies	Variants without impact on splicing	120 (38 / 82; 31.7%)

**Fig. 2:**
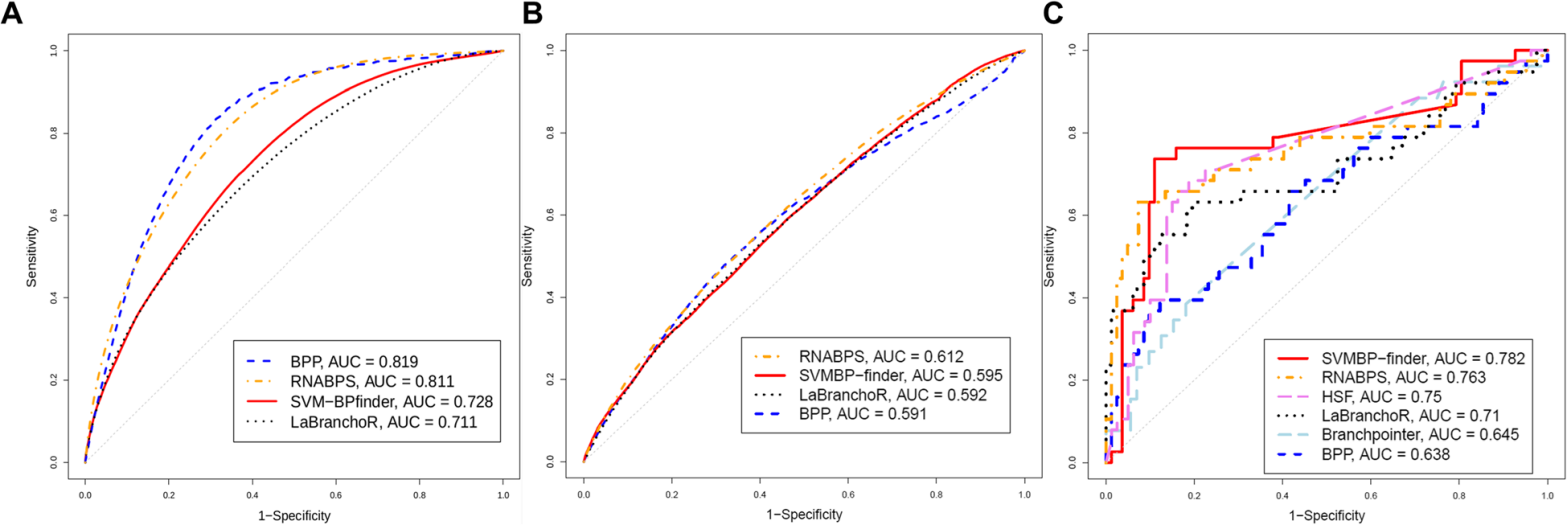
ROC curves of the bioinformatics scores. For each possible score threshold, sensitivity and specificity were plotted. **a**. The detection of branch points from the set of Ensembl acceptor splices sites (*n* = 114,868,082) of BPP, SVM-BPfinder, LaBranchoR and RNABPS scores. **b**. The detection of branch points from the alternative 3’ss by the SVM-BPfinder, BPP and LaBranchoR (*n* = 103,972). **c**. The delta scores of HSF, SVM-BPfinder, BPP, Branchpointer, LaBranchoR and RNABPS to class variants (*n* = 120)

We first retrieved 264,787 Ensembl 3’ss from the Ensembl data. Adding to these 3’ss, 114,603,295 random AGs were used as control data (see the “Methods” section for details). Thus, we collected 114,868,082 3’ss. ROC curve analysis was then performed for SVM-BPfinder, BPP, LaBranchoR and RNABPS on the set of Ensembl 3’ss, as illustrated in Fig. 2a. Table 3 shows the levels of accuracy, sensitivity, specificity, positive predictive value (PPV) and negative predictive value (NPV) derived from these ROC curve analyses. In terms of the area under the curves (AUC), the score provided by BPP exhibited the best performance (AUC = 0.818). However, Branchpointer presented the highest performances with an accuracy of 99.49% and PPV of 30.06%. Thus, Branchpointer was the most stringent of the bioinformatic tools for detecting putative BPs upstream of Ensembl 3’ss. Indeed, SVM-BPfinder, BPP, LaBranchoR and RNABPS detected putative BPs for each Ensembl 3’ss and random AGs. For these 4 tools, the best accuracy to distinguish Ensembl 3’ss from random AGs was reached by BPP (75.23%). Overall, 74,539,834 3’ss had a BP predicted by at least one tool. The maximum overlap of predicted BPs was observed between LaBranchoR and RNABPS (28.63%; 21,337,483/74,539,834 3’ss) (Additional file 1: Figure S4). The percentage of 3’ss with BP predicted by the five tools was 0.15% (111,937/74,539,834). Seventy-five percent (83,892/111,937) of these 3’ss were Ensembl 3’ss (Additional file 1: Figure S5).

**Table 3:** Performance of tools derived from contingency table with Ensembl dataset (*n* = 114,868,082)

	SVM-BPfinder	BPP	Branchpointer	LaBranchoR	RNABPS
Cutoff	0.706	5.384	–	0.653	0.653
TP	166,135	198,708	252,967	171,511	193,430
FP	36,526,998	28,315,554	583,920	40,370,908	30,878,750
TN	72,145,972	86,003,592	114,019,375	74,232,290	83,724,448
FN	84,113	65,422	11,820	93,276	71,357
Missing data	5,944,864	284,806	0	97	97
AUC	0.728	0.819	–	0.711	0.811
Accuracy	66.39%	75.23%	99.48%	64.77%	73.06%
Sensitivity	66.39%	75.23%	95.54%	64.77%	73.05%
Specificity	66.39%	75.23%	99.49%	64.77%	73.06%
PPV	0.45%	0.70%	30.23%	0.42%	0.62%
NPV	99.88%	99.92%	99.99%	99.87%	99.91%

*TP* (True Positive), *FP* (False Positive), *TN* (True Negative), *FN* (False Negative), *AUC* (Area Under the Curve), *PPV* (Positive Predictive Value), *NPV* (Negative predictive value)

Among the alternative junctions of whole transcriptome analysis, 51,986 alternative 3’ss were identified (see the “Methods” section for details and Additional file 1: Figure S6), to which we added the same number of control 3’ss. In all, we had 2 subsets of 51,986 (103,972) acceptor sites for whole transcriptomic data (Additional file 2: Table S1). The SpliceLauncher analysis revealed that 99.5% of splicing junctions (51,703/51,988, data not shown) did not have a significant expression difference across the different cell culture conditions and the different variants. The relative expression of the alternative 3’ss appeared to follow a log-normal distribution (Shapiro-Wilk *p*-value = 0.09 and Additional file 1: Figure S7). From these data, Branchpointer outperformed all tested tools for detecting putative BPs (Table 4). Indeed, the AUC of the three tools, SVM-BPfinder, BPP, LaBranchoR and RNABPS, did not perform above 0.612 (RNABPS) (Fig. 2b). Branchpointer showed the best accuracy of 65.8% on the alternative splice sites. Furthermore, this tool demonstrated a similar specificity with the Ensembl and RNA-seq data, 99.6 and 99.5%, respectively. However, on the whole transcriptome data, the sensitivity decreased by more than 60% (from 95.5 to 32.1%) (Table 3 and Table 4). The alternative 3’ss and control 3’ss had BPs predicted by at least one of the tools in 91.2% (94,806/103,972). The maximum overlap was observed between the four tools SVM-BPfinder, BPP, LaBranchoR and RNABPS (7227/94,806 3’ss). More than 95% of 3’ss with a BP predicted only by Branchpointer were alternative splice sites (Additional file 1: Figure S8). In a paired comparison, the two tools LaBranchoR and RNABPS displayed a maximum overlap of 34.57% (32,777/94,806 3’ss) with common BPs (Additional file 1: Figure S4).

**Table 4:** Performance of the bioinformatics tools on the alternative acceptor splice sites (*n* = 103,972)

	SVM-BPfinder	BPP	Branchpointer	LaBranchoR	RNABPS
Cutoff	0.76997	5.55569	–	0.66239	0.6962
TP	28,990	29,953	16,671	29,346	29,320
FP	22,608	22,033	206	22,640	21,894
TN	29,132	29,953	51,780	29,346	30,092
FN	22,499	22,033	35,315	22,640	21,274
Missing data	743	0	0	0	1482
AUC	0.595	0.591	–	0.592	0.612
Accuracy	56.3%	57.6%	65.8%	56.4%	57.9%
Sensitivity	56.3%	57.6%	32.1%	56.4%	57.9%
Specificity	56.3%	57.6%	99.6%	56.4%	57.9%

*TP* (True Positive), *FP* (False Positive), *TN* (True Negative), *FN* (False Negative), *AUC* (Area Under the Curve)

**Fig. 3:**
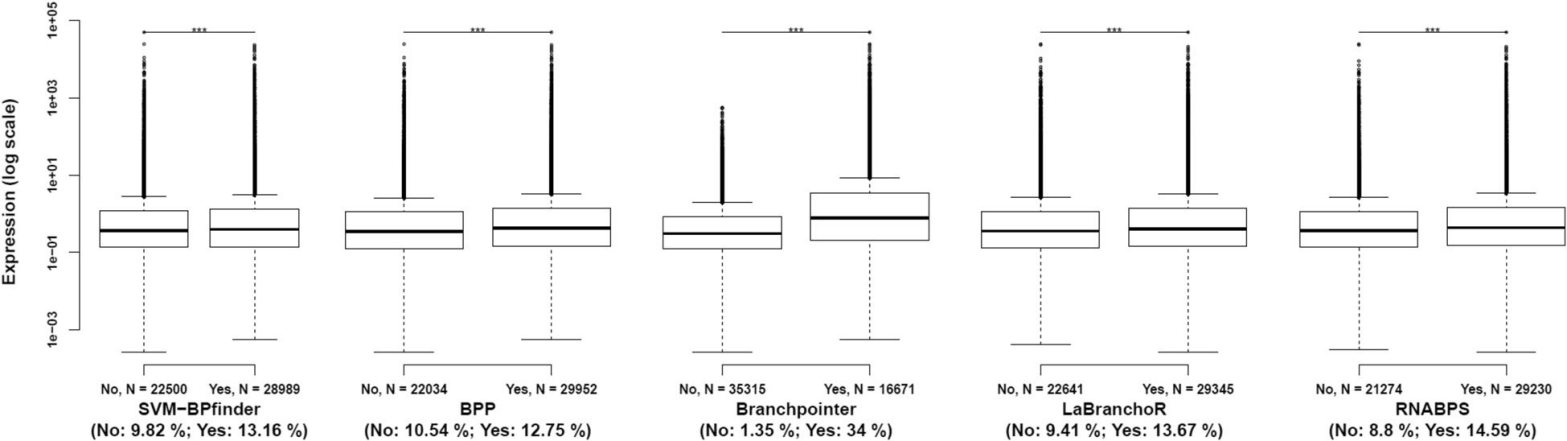
Expression of 3’ss according the presence or not of predicted branch point by the bioinformatics tools, from RNA-seq data (*n* = 51,986 3’ss). ***: *p*-value (Student test) <2e-16. In brackets, the average expression between the two groups

We compared the expression of alternative sites, from RNA-seq data, with and without the presence of a putative BP predicted by the bioinformatic tools (see the “Methods” section for details). This analysis revealed that 3’ss with a predicted BP were significantly more expressed than 3’ss without a predicted BP, regardless of the bioinformatics tool (Fig. 3). The greater difference of expression was observed for Branchpointer. The average expression was 34.00 and 1.35%, for alternative 3’ss with Branchpointer-predicted BP or not, respectively. In the subgroup of 3’ss with a predicted BP, the Branchpointer score was not correlated with the expression of these sites (R^2^ = 0.00001, *p*-value = 0.24). The other bioinformatics tools presented a weak correlation between their score and the expression (Additional file 1: Figure S9). Among SVM-BPfinder, BPP, LaBranchoR and RNABPS, the best correlation was obtained with RNABPS (determinant coefficient (R^2^) = 0.0062, *p*-value = 4.14 × 10^− 70^).

### Bioinformatic prediction of splicing effect for variants in the branch point area

**Fig. 4:**
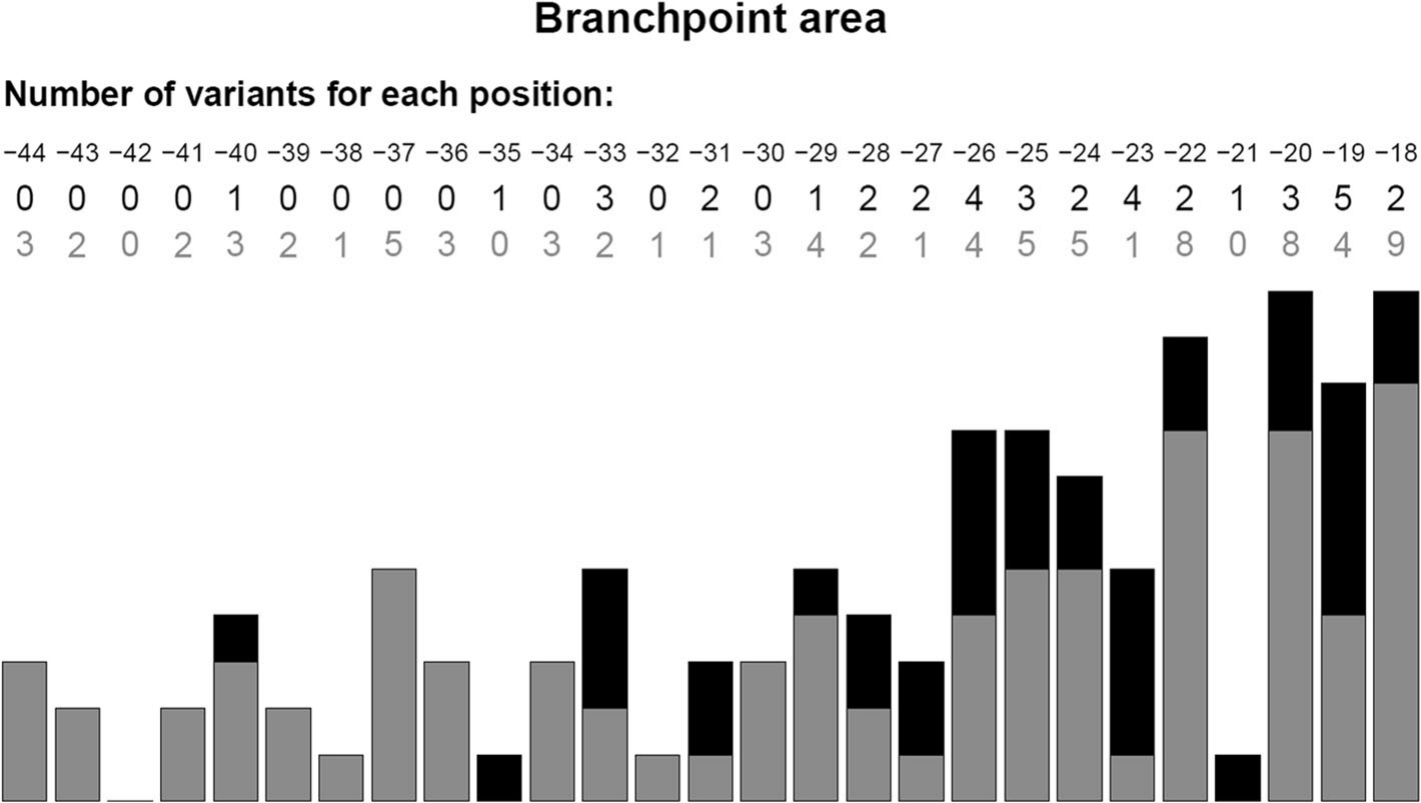
Distribution of intronic variants in the branch point area (− 18 to − 44) experimentally tested for their impact on RNA splicing (*n* = 120). Positions are relative to the nearest reference [3]’ss. In black variants that altered RNA splicing. In grey, variant without effect

The last set of data was a collection of experimentally characterized potentially spliceogenic variants mapping within BP areas (see the “Methods” section for details), *n* = 120 variants among 86 introns in 36 different genes (Table 2 and Additional file 3: Table S2). Part of this collection was obtained from unpublished data (*n* = 62 variants). From the 120 variants, 38 (31.7%) were found to induce splicing alteration, and were therefore considered as spliceogenic, whereas 82 (68.3%) did not show splicing alterations under our experimental conditions. Fig. 4 indicates the repartition of the 120 variants within the corresponding BP areas and their impact on RNA splicing. The 38 spliceogenic variants were identified in 30 different introns; 22 variants induced exon skipping, 10 variants caused full intron retention and six remaining variants activated the use of another cryptic 3’ss located up to 147 nt upstream of the 3’ss and 38 nt downstream of the initial acceptor site (Additional file 3: Table S2).

After the prediction of BPs for each intron affected by the variants, we analyzed the distribution of each variant according to the position of the predicted BP (Additional file 1: Figure S10). First, we assayed the different size motifs to classify variants (see the “Methods” section for details). The best common motif was the 4-mer starting 2 nt upstream of the A and 1 nt downstream (Additional file 1: Figure S11), that corresponds to the motif TRAY. For this size motif, BPP presented the best accuracy with 89.17% and LaBranchoR had the lower performance with an accuracy of 78.33% (Table 5). Branchpointer did not predict a BP for the intron 24 of *BRCA2* gene causing a missed data point, corresponding to *BRCA2* c.9257-18C > A variant.

**Table 5:** Classification of variants according their position in the predicted branch point (*n* = 120) (Motif 4-mer: TRAY)

	SVM-BPfinder	BPP	Branchpointer	LaBranchoR	RNABPS
TP	24	32	32	27	30
FP	6	7	12	15	12
TN	76	75	69	67	70
FN	14	6	6	11	8
Accuracy	83.33%	89.17%	84.87%	78.33%	83.33%
Sensitivity	63.16%	84.21%	84.21%	71.05%	78.95%
Specificity	92.68%	91.46%	85.19%	81.71%	85.37%

*TP* (True Positive), *FP* (False Positive), *TN* (True Negative), *FN* (False Negative)

As shown in Additional file 1: Figure S10, variants affecting splicing were mostly located at putative branch point positions 0 (the predicted branch point A) and − 2 (the T nucleotide 2 nt upstream of the branch point A itself). BPP pinpointed the highest number of spliceogenic variants in these positions. More precisely, splicing anomalies were detected for all of the ten variants occurring at position − 2, and for 15 out of 18 variants predicted to be located at the branch point A. The three remaining variants predicted by BPP to alter the branch point A position (*BRCA1* c.4186-41A > C, *MLH1* c.1668-19A > G and *RAD51C* c.838-25A > G), and not experimentally validated, were also predicted to alter a BP adenosine by SVM-BPfinder while Branchpointer and LaBranchoR placed these variants outside BP motifs.

Next, we assessed the discriminating capability of each tool, including HSF, by calculating delta scores, to identify splicing defects from BP variants (Fig. 2c). In terms of delta score, SVM-BPfinder outperformed the other tools with an AUC of 0.782. From this ROC analysis, we identified an optimal decision threshold (see the “Methods” section for details) of − 0.136, i.e. the variants were predicted as spliceogenic if the variant score was less than 13.6% of the wild-type score. The performances achieved with this threshold are reported in Table 6. SVM-BPfinder reached the maximum accuracy of 81.67%.

**Table 6:** Contingency table of variant according to the variation score, *n* = 120 variants

	HSF	SVM-BPfinder	BPP	Branchpointer	LaBranchoR	RNABPS
Cutoff	−0.0378	−0.136	− 0.0006	−0.0003	− 0.0194	−0.0304
TP	27	29	22	10	25	27
FP	18	13	31	13	25	20
TN	62	69	51	59	57	62
FN	12	9	16	16	13	11
AUC	0.750	0.782	0.638	0.645	0.710	0.763
Accuracy	75.4%	81.67%	60.8%	70.4%	68.3%	74.2%
Sensitivity	71.1%	76.32%	57.9%	38.5%	65.8%	71.1%
Specificity	77.5%	84.15%	62.2%	81.9%	69.5%	75.6%

*TP* (True Positive), *FP* (False Positive), *TN* (True Negative), *FN* (False Negative), *AUC* (Area Under the Curve)

The achievement of cross-validation, from the logistic regression model, highlighted the performance of combination of the BPP and Branchpointer tools (see the “Methods” section for details). This model was to infer variants as spliceogenic if they occurred within a TRAY 4-mer BP motif predicted by both BPP and Branchpointer. Although this combination was mostly found in the 1000 simulation, this model appeared in only 26% of these simulations (see Additional file 1: Figure S12). The likelihood ratio test between this model and a model with only the BPP tool was not systematically significant, with 60.1% of simulations having *p*-value above 1%. This approach also showed that for a variant in intron with different and non-overlapping predicted BP sites by BPP and Branchpointer, the model could not provide prediction of potential spliceogenicity. We continued the cross-validation without the positions of predicted BP for all tools except BPP. However, the delta scores of other tools did not improve the model, as the majority of simulations converging to BPP-alone model (Additional file 1: Figure S13). Thus, the analysis revealed that the position of the BPs predicted by BPP alone was the optimal model.

## Discussion

In this study we benchmarked 6 different tools for their ability to detect either a physiological BP, or a variant-induced BP alteration. From Ensembl data, Branchpointer showed the best performance with an accuracy of 99.48%. This highlighted the interest of the machine learning approach compared to support vector machine and mixture models used in the development of SVM-BPfinder and BPP, respectively. The deep learning tools, LaBranchoR and RNABS showed the maximum number of common predicted BPs from Ensembl (28.63%) and from RNA-seq (33.57%) data. Indeed, these two tools are both based on the same deep learning approach (bidirectional long short-term memory) and used the same sequence length (70 nt) as input [20, 21]. By comparison, RNABPS employed a dilated convolution model explaining and showed an improvement of prediction compared to LaBranchoR (73.06% against 64.77% of accuracy) using the Ensembl data (Table 3). One would have expected that RNABPS and LaBranchoR, using a deep learning approach, should have performed equal or above to Branchpointer. However, these tools reached an accuracy of 73.06% (RNABPS) and 99.48% (Branchpointer) using the Ensembl data (Table 3). To explain the results, we propose two hypotheses. Firstly, the three tools (Branchpointer, LaBranchoR, and RNABPS) used the collection of experimentally-proven collection of BPs published by Mercer and Coll [10].. Whereas Branchpointer used a large collection of negative BPs as control data (52,843 true BPs and 878,829 false BPs) [19]. Furthermore, LaBranchoR, and RNABPS were only trained on the 70 nt upstream of 3’ss with known BPs, 27,711 3’ss and 71,753 3’ss respectively. BPP also was not trained with a collection of false BPs, and SVM-BPfinder was only trained on putative BP. Thus, on our Ensembl data, Branchpointer is more powerful to detect the BPs among the background noise, *i. e.* the unexpected BPs sequences with random AGs (see the “Methods” section for details). Secondly, Branchpointer takes into account the structure of transcripts unlike LaBranchoR and RNABPS. Indeed, Branchpointer considers only the prediction of BPs occurring in − 44 and − 18 upstream of 3’ss.

The relative expression of junctions was significantly correlated to the bioinformatic scores. However, these correlations remain weak, with a maximum coefficient of determination (R^2^) of 0.0062 for RNABPS. Added to this, even if Branchpointer had shown the best performance, the sensitivity of Branchpointer decreased by almost 60% (95.54 to 32.1%) between the Ensembl and RNA-seq data. Alternative 3’ss, without Branchpointer prediction, were expressed at relative low levels. Branchpointer was trained on the high-confident BPs and the low confidence BPs were considered as negative [19]. This issue highlighted the limit of detection of Branchpointer, for the weakly used 3’ss or the less conserved BPs. The performance of Branchpointer confirms the importance of the BP in 3’ss definition, but does not explain the expression level of these 3’ss. This last point highlights the complexity of splicing that does not only depend on the 5’ss, 3’ss and the BP. To illustrate this complexity, a recent study was published [24] demonstrating the MMSplice tool which gathers several features from intronic and exonic pre-mRNA sequences. This tool was assayed on the Vex-seq data [25] which consists of 2059 human genetic variants in and around 110 exons. For each variant the authors displayed the percentage of exon inclusion by minigene splicing assays. The correlation between this percentage and the MMSplice score reached an R^2^ of 0.48 (=0.69^2^). Despite accounting for both set of splicing motifs and the BP motifs, more than 50% of expression variability of exon inclusion remained unexplained by the predictions.

To investigate potential spliceogenic variants occurring in the BP area, we gathered a large collection of 120 human variants (62 unpublished), with their corresponding in vitro RNA data. From our analysis, the best prediction strategy was to consider the variant as impacting the splicing if it is located in the BP motif. With this strategy the best score was obtained by BPP with an accuracy equal to 89.17%. We observed that only 31.7% (38/120) of variants altered the splicing in the BP area while 82.05% (32/39) alter splicing in the BPP-predicted BP motif with a sensitivity of 84.21%. These results demonstrate the potential of BPP for prioritizing variants occurring in this region for molecular diagnostic laboratories. From our dataset, we first determined, that the 4-mer **T**R**A**Y in the BP motif was the most impacted by variants. A variant occurring in this motif has a high probability to alter splicing. In our work, this probability was 82% with BPP tool while the proportion of variants affecting the splicing outside this motif was 7.4%. This bioinformatic tool takes into account several features in and around the 4-mer motif. Variants outside the BP motif can modify the score of BPs, although having a weak risk splicing alteration. Thus variants can wrongly affect the score. Indeed, 37 (45.7%) variants occurring outside this 4-mer motif decreased the BPP score whereas only 4 (10.8%) of these variants impacted splicing. Therefore, we excluded the delta score used to predict the BP alteration by a variant. The alignment of variants on the BPP-predicted BP revealed that the most spliceogenic variants were localized at the nucleotide position 0 (A) and − 2 (T) of the BPs. The highly conserved di-nucleotides at the position 0 and − 2 [26] were critical to the BP recognition. These observations also suggest that BPP-detected BPs are functional. The variant collection did not take into account the capability of BP detection among the background noise, the variants occurring in area (− 18; − 44) with an expected branch point. In this context, BPP reached better performance than the other tools. On the other hand, the high specificity to remove background noise penalized Branchpointer on the variant collection. For an example in the intron 24 of *BRCA2* gene the non-detection of a BP by Branchpointer hindered the prediction of spliceogenic variants (Additional file 3: Table S2). The first study of a large collection of BPs identified the presence of redundant BPs [10]. We also observed that variants altering high BP scores, as predicted by BPP induced splicing alterations in the vast majority of cases (82%). Among the introns (*n* = 86) studied in this work, the potential redundancy of BPs was not sufficient to allow natural splicing to be completely restored. In our analyses, we did not focus on the quantitative effect of splicing, due to the diversity of RNA in vitro studies. Among the data generated in this study, eight of the variants that impacted splicing were assessed using minigene assays. In these condition, these variants produced both the natural and aberrant transcripts, i.e. they had a partial effect (data not shown). The presence of redundant BPs could explain this partial effect. However, this was beyond the scope of the present benchmarking study and will need to be explored in future studies. We observed that sequence alteration of BPs induced not only exon skipping but also intron retention and the use of new distant 3’ss. Thus, these predictions will permit the prioritization of RNA in vitro studies rather than determine the exact effect on splicing. The combination of BPP and Branchpointer, slightly improved prediction of BP position. Moreover, for introns with non-overlapping BPP and Branchpointer-predicted BP positions, the model will not draw a conclusion regarding the spliceogenicity of a variant. From a practical view point, the combination of scores makes the predictions of BPs less accessible.

The accessibility of the tools represents a technical limit to the analysis of BP. Indeed, HSF, SVM-BPfinder and RNABPS have a Graphical User Interface web page for non-bioinformatician users. However, LaBranchoR and BPP score calculation was only accessible by a python script. LaBranchoR also offers a list of potential BPs predicted by the tool and visualization via the UCSC Genome Browser [27]. Branchpointer is only accessible by an R package and needs the installation of several other libraries. Due to machine learning calculation, this tool also has the longest run-time. The score calculation for the Ensembl data set (*n* = 114,868,082) with a Linux machine AMD® Ryzen 7 pro 1700 eight-core processor, 8 Gb of RAM with multiprocessing way (6 at the same time) took more than two weeks, instead of a couple of days for SVM-BPfinder. Added to this, HSF tool did not allow an analysis of batches of the BP and so makes the analysis difficult of variant obtained from next-generation sequencing.

## Conclusion

Our study spotlighted the requirement to distinguish two issues, the capacity to detect a real BP and the capacity to predict the splicing alteration at BP level. Branchpointer exhibited the best performance to detect a real BP from our Ensembl and RNA-seq data. For research purposes, Branchpointer facilitates the study of alternative transcripts by predicting the most likely used alternatively spliced 3’ss. However, the BPP-predicted BPs were more efficient to predict the impact of variants on BP usage. Furthermore, BPP was able to predict 4-mer BP motifs, with an accuracy of 89.17%. Using a large collection of human variants (*n* = 120) with associated RNA in vitro splicing data, we confirm the advantage of studying the BP area ([− 44–18] intronic positions) for application to molecular diagnostics. As the next generation sequencing era increases the number of variants detected across exonic and intronic regions, we show how these BP prediction tools can assist the diagnostician by prioritizing variants for in vitro RNA studies.

## Methods

### Sets of data

The Ensembl dataset contains the coordinates of a large collection of transcripts [28], with more than 200,000 human transcripts (download June 28th 2018). We extracted the position of exons for each described transcript then we deduced the coordinates of splice sites. As random data, we took all AG sequences found in each transcript sequence, named hereafter random AGs. For each 3’ss, the genomic coordinates were annotated according to the hg19 genome assembly.

We defined as alternative splice sites all 3’ss identified from RNA-seq data that were not described in the transcripts from the RefSeq dataset [29] The alternative 3’ss were obtained from our in-house RNA-seq analyses. The read count mapped on these last RefSeq 3’ss served as a reference to calculate the relative expression of the alternative 3’ss. Whole transcriptome RNA-Seq experiment was performed on 72 RNA samples corresponding to lymphoblastoid cell lines (LCLs) from four control individuals and eight patients with pathogenic variants in *TP53* or in the *BRCA1/2* genes, treated and untreated with bleomycin or doxorubicin, and performed in triplicate. Ribosomal RNA was depleted using the NEBNext® rRNA Depletion Kit (Human/Mouse/Rat) (NEB, Ipswich, MA, USA) and libraries were produced using the NEBNext® Ultra™ RNA Library Prep Kit for Illumina® (NEB). 2x75b paired-end sequencing was performed on an Illumina NextSeq500 yielding an average of 50 million paired reads per sample. Reads were aligned on the Ensembl reference genome GRCh37 release 75 (ftp://ftp.ensembl.org/pub/) using STAR v2.5.3a tool (Spliced Transcripts Alignment to Reference) [30] and counting was performed using FeatureCounts tool v1.5.2 [31]. To avoid the impact of cell culture condition and the effect of variants on the expression of alternative 3’ss, we selected alternative splice sites observed in more than six samples. Each condition and variant was analyzed in triplicate. Then with this threshold we removed any splicing junctions linked to the particular conditions or variants. The expression of alternative splice sites was calculated as follows:

$$ {\%}_{expression}=\frac{{read\ count}_{alternative\ site}}{{read\ count}_{physiological\ site}}\times 100 $$

The read count corresponded to the number of reads mapping on exon junctions and the physiological site was defined as the nearest splice site, described in RefSeq, and same type of alternative splice site. The detailed model of the alternative splicing was proposed by Davy and collaborators [32].. The percentage of expression permitted the estimation of a weighted expression that allows for the versatility of splicing events. The tool SpliceLauncher v2 was used to perform the calculations and also to detect abnormal splicing junction expression [33].

As control data for the set RNA-seq data, we took any 3’ss that had a MaxEntScan [34] score higher than 0 but was not identified in the RNA-seq data or in the RefSeq database. We called its control 3’ss. Among these control 3’ss, we randomly selected 3’ss so that the number of control 3’ss was equivalent to the number of alternative 3’ss.

The last set of data was a collection of potential spliceogenic variants, characterized by experimental RNA studies, occurring in the BP area (from − 18 to − 44 relative to the 3’ss) of 36 genes. Briefly, this dataset included RT-PCR data obtained from (i) minigene-based splicing assays (by Inserm U1078 and by Inserm U1245 teams), (ii) RNA extracted from lymphoblastoid cell lines treated/untreated with puromycin, (iii) RNA extracted from blood collected into PAXgene tubes (Qiagen), (iv) RNA extracted from stimulated T lymphocytes provided by the French Splice Network of the Unicancer Genetic Group. Controls (samples a without a variant in the BP area) were systematically included in these experiments [35]. During the collection, we excluded any variant that altered splicing by creation or reinforcement of a cryptic or de novo consensus splice site. Owing to the fact that the data were heterogeneous in term of analyses and submitters, we did not take into account the quantitative information of splicing alteration. Thus, we pooled together variants having a partial or total effect on splicing.

### Assessment of bioinformatics tools

Six BP-dedicated in silico tools were tested: HSF v3.1, SVM-BPfinder, BPP, Branchpointer v3.8, LaBranchoR and RNABPS (Table 1). On the other hand, we were confronted with an inaccessible tool, the BPS predictor [36], at the time of this work, so it was excluded from this study. HSF v3.1 did not allow browsing of a wild-type sequence to detect potential BPs, and gave only the score change of a variant. For the other tools, we used the standalone versions that were: python scripts for SVM-BPfinder, BPP and LaBranchoR and R package for Branchpointer. For the tools SVM-BPfinder, BPP and Branchpointer, we narrowed the browsed sequence by these 3 tools to include 1 to 200 nt upstream of the 3’ss. LaBranchoR and RNABPS need a 70 nt long sequence, and the browsed sequence was the 70 last nt of the intron. The use of the different tools did not need parameter settings, except for SVM-BPfinder. The tool required definition of the number of bases at the 3′ end of the input sequences that will be scanned, and the distance in nucleotides allowed between the branch point A and the 3’ss. We used the default values, where the scanned sequence length was 100 nt and the distance between the branch point A and the 3’ss was 15 nt.

Receiver operating characteristic (ROC) analysis was performed for the tools generating continuous scores (SVM-BPfinder, BPP, LaBranchoR, RNABPS). From each ROC curve, we determined an optimal decision threshold defined as the threshold with the minimal difference between the sensitivity and specificity. Branchpointer displayed only BPs with high confidence level, so we processed directly to a contingency table between the true 3’ss and the control AG with predicted BPs (Additional file 1: Figure S1).

From the RNAseq data, the relative expression of alternative 3’ss was studied according the BP-predictions of bioinformatic tools. We compared the expression between the two groups: 3’ss with predicted-BP and 3’ss without predicted-BP. The Student test was used under the hypothesis that the relative expression follows a log-normal distribution. The hypothesis of the log-normal distribution was assessed by a Shapiro-Wilk test, and was performed on the logarithm of expression.

To study the effect of nucleotide variants on RNA splicing, we considered two questions, i) Is the variant located in a putative BP? and ii) Does the variant decrease the score of the putative BP? Given that the first question concerns a binary variable, we used contingency tables to compare the performance of the different tools. We started by using five out of the six tools (exclusion of HSF) to define a list of predicted BPs in the browsed sequences from each intron that are affected by the variants in our dataset. Next, we took only one BP with the highest score per intron, for each tool. To determine whether the variant was located in the motif of the predicted BP, we assayed different motif sizes from 1 nt (corresponding to the branch point A) up to 7-mer around the A, i.e. the 3 nt on either side of A. The 7-mer motifs corresponded to the length of position weight matrices used by the majority of the tools (Fig. 1). We established the optimal motif size as having the best compromise of sensitivity and specificity across all tools. The second question involved the calculation of a delta score defined as follows:

$$ Delt{a}_{score}=\frac{Scor{e}_{variant\ site}- Scor{e}_{wildtype\ site}}{Scor{e}_{wildtype\ site}} $$

This delta score did not necessarily imply that wild-type and variant scores were from the same BP site. Different examples are illustrated in Additional file 1: Figure S2. On this delta score we performed ROC curve analyses and then defined an optimal decision threshold to classify the variants.

### Evaluation of the score combination

To determine the optimal score combination, we used a logistic regression. This model provided a probability that the variant alters RNA splicing depending on the information given by the bioinformatic scores. We performed a cross-validation, with two thirds of the data being used as training set and the remaining data as validation set. The data was allocated at random and this step was repeated 1000 times. On the training set, we executed a step-by-step variable selection (stepwise). On the validation set, the performances of the probability given by the model were evaluated by a ROC curve analysis.

## Supplementary information

**Additional file 1: Figure S1.** Workflow to compare bioinformatics tools on Ensembl and RNA-seq data for the predictions of branch point (BP). **Figure S2.** The different ways that a variant may alter the branch point score. **Figure S3.** Running time of the four tools SVM-BPfinder, BPP, Branchpointer, and LaBranchor. **Figure S4.** Paired comparison of the five tools from the Ensembl data and from the RNA-seq data. **Figure S5.** The overlap of natural 3′ ss (True Calls) and controls AG (False Calls) from Ensembl data. **Figure S6.** Splicing junctions filtered out from RNA-seq data. Alt 3’ss: alternative acceptor splice sites. **Figure S7.** The distribution of the relative expression of alternative 3’ss. **Figure S8.** The overlap of alternative 3′ ss (True Calls) and controls AG (False Calls) from our RNAseq data. **Figure S9.** Correlation between the scores (SVM-BPfinder, BPP, Branchpointer, LaBranchoR, RNABPS) and the expression of alternative 3’ss. **Figure S10.** Repartition of variants (*n* = 120) according their position relative to the predicted branch point. **Figure S11:.** Determination of optimal motif (YTRAYNN) length to predict splicing alteration, n = 120 variants. ACC: Accuracy, Pos: relative position in branch point motif, Se: Sensitivity, Sp: Specificity. **Figure S12.** Cross-validation (1000 times) to select the optimal model to predict branch point alteration. **Figure S13.** Cross-validation (1000 times) to select the optimal model to predict branch point alteration without the positions of predicted BP for all tools except BPP.

**Additional file 2 Table S1**. Collection of alternative acceptor splice site (3’ss) and controls 3’ss (*n* = 103,972), from RNA-seq data

**Additional file 3 Table S2**. Collection of variants used to compare the branch point predictions (*n* = 120)

## Data Availability

The scripts used for the evaluation of algorithms’ performance are available at https://github.com/raphaelleman/BenchmarkBPprediction. The set of Ensembl 3′ ss and control AG were constructed with the dataset download from Ensembl [28] (https://www.ensembl.org/index.html). The alternative 3’ss, from RNAseq data, and controls were shown in supplemental (Additional file 2: Table S1). All variants reported in this study were in supplemental information (Additional file 3: Table S2).

## Abbreviations

3’ss: Acceptor splice site
5’ss: Donor splice site
AGEZ: AG exclusion zone algorithm
AUC: Area under the curve
BP: Branch point
BPP: Branch point prediction
HSF: Human splicing finder
LaBranchoR: Long short-term memory network branchpoint retriever
LCL: Lymphoblastoid cell line
LSTM: Long short-term memory network
MMSplice: Modular modeling of splicing
mRNA: RNA messenger
NPV: Negative predictive value
nt: Nucleotide
PPV: Positive predictive value
pre-mRNA: Pre-mature mRNA
RNA: Ribo-nucleic acids
RNABPS: RNA Branch Point Selection
RNAseq: RNA sequencing
STAR: Spliced transcripts alignment to reference
UCSC genome browser: University of california santa cruz
Vex-seq: Variant exon sequencing
