# Supplementary material for: Assessment of branch point prediction tools to predict physiological branch points and their alteration by variants
Source: BMC Genomics. 2020 Jan 28;21:86. doi: 10.1186/s12864-020-6484-5 (PMC6988378; doi:10.1186/s12864-020-6484-5)
Supplement: Supplementary file 1 — Additional file 1: Figure S1. Workflow to compare bioinformatics tools on Ensembl and RNA-seq data for the predictions of branch point (BP). Figure S2. The different ways that a variant may alter the branch point score. Figure S3. Running time of the four tools SVM-BPfinder, BPP, Branchpointer, and LaBranchor. Figure S4. Paired comparison of the five tools from the Ensembl data and from the RNA-seq data. Figure S5. The overlap of natural 3′ ss (True Calls) and controls AG (False Calls) from Ensembl data. Figure S6. Splicing junctions filtered out from RNA-seq data. Alt 3’ss: alternative acceptor splice sites. Figure S7. The distribution of the relative expression of alternative 3’ss. Figure S8. The overlap of alternative 3′ ss (True Calls) and controls AG (False Calls) from our RNAseq data. Figure S9. Correlation between the scores (SVM-BPfinder, BPP, Branchpointer, LaBranchoR, RNABPS) and the expression of alternative 3’ss. Figure S10. Repartition of variants (n = 120) according their position relative to the predicted branch point. Figure S11:. Determination of optimal motif (YTRAYNN) length to predict splicing alteration, n = 120 variants. ACC: Accuracy, Pos: relative position in branch point motif, Se: Sensitivity, Sp: Specificity. Figure S12. Cross-validation (1000 times) to select the optimal model to predict branch point alteration. Figure S13. Cross-validation (1000 times) to select the optimal model to predict branch point alteration without the positions of predicted BP for all tools except BPP. [file 12864_2020_6484_MOESM1_ESM.docx]

# **SUPPLEMENTARY INFORMATION: ‘Assessment of branch point prediction tools to predict physiological branch points and their alteration by variants’**

The supplementary Table S1 and Table S2 are provided separately in Excel format.

# **Figure S1:** Workflow to compare bioinformatics tools on Ensembl and RNA-seq data for the predictions of branch point (BP).


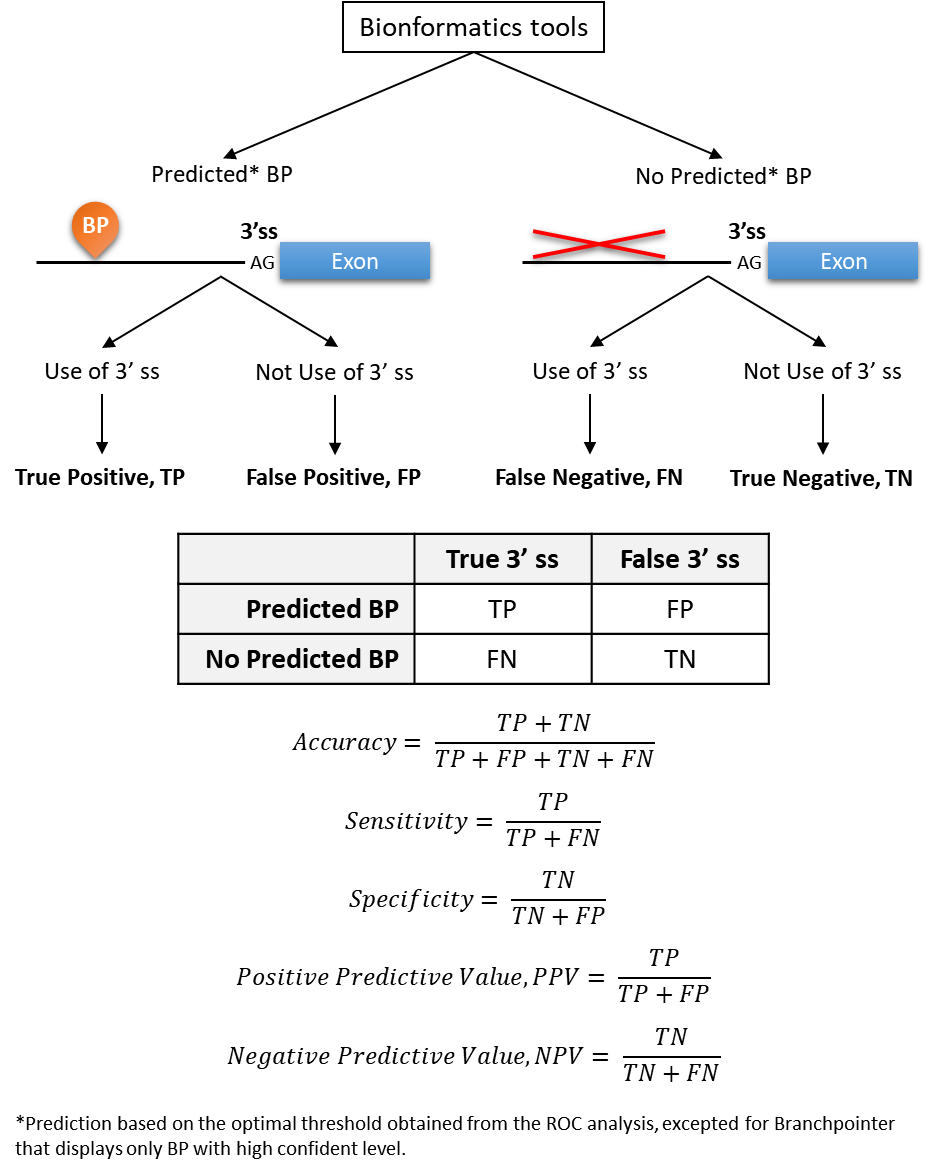


# **Figure S2:** The different ways that a variant may alter the branch point score.


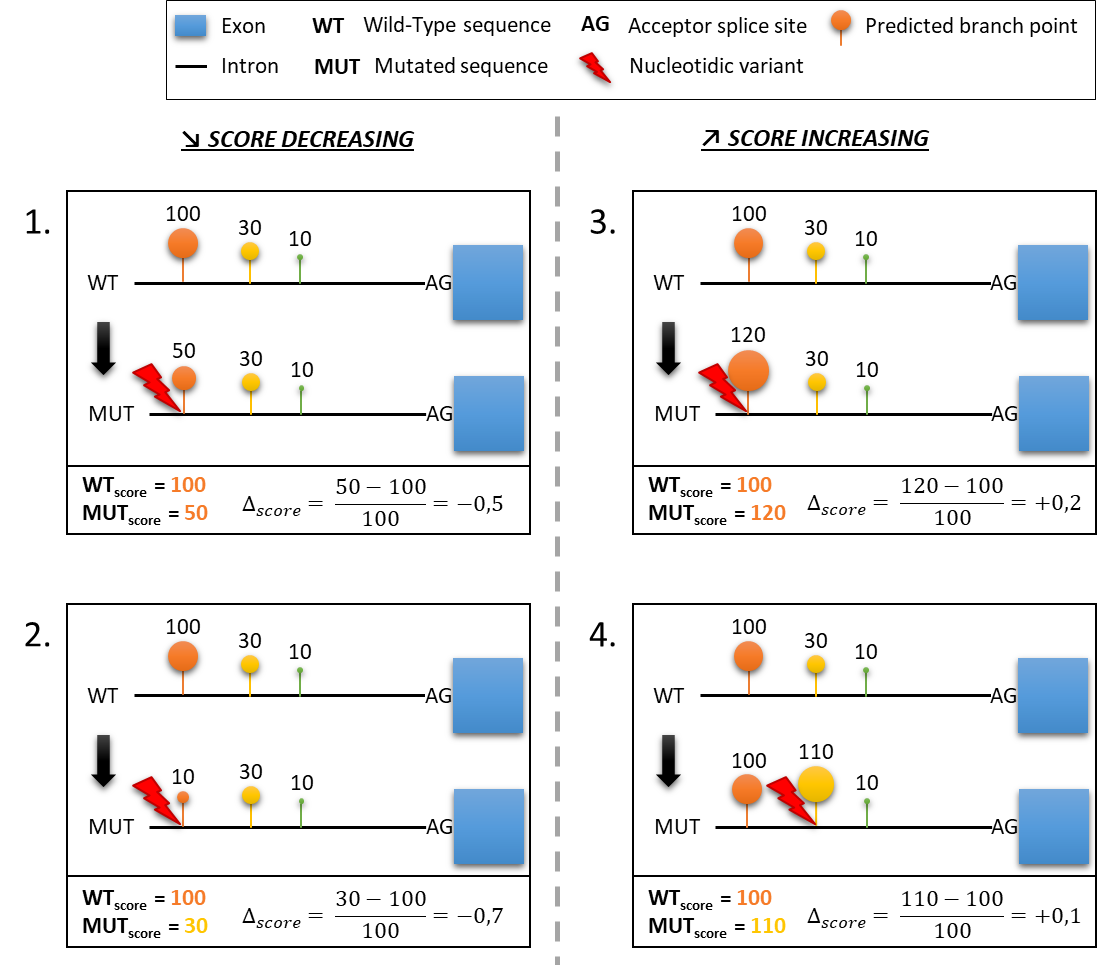


# **Figure S3:** Running time of the four tools SVM-BPfinder, BPP, Branchpointer, and LaBranchor. Comparison performed on a AMD Ryzen 7 PRO 1700 Eight-Core Processor 3.00 GHz (16 Go) and on a sample of 1000 sequences as input. The tools HSF had not running time because unavailable for batch analysis and the RNABPS calculation score was performed by Tayara Hilal. Then for the two tools the running time was not calculated.

# **Figure S4:** Paired comparison of the five tools from the Ensembl data and from the RNA-seq data

From the Ensembl data

| N = 74,539,834 | SVM-BPfinder | BPP | Branchpointer | LaBranchoR | RNABPS |
| --- | --- | --- | --- | --- | --- |
| SVM-BPfinder | 49.24 % (36,700,973) | 16.26 % (12,117,585) | 00.54 % (404,453) | 21.30 % (15,873,664) | 20.25 % (15,096,146) |
| BPP |  | 38.33 % (28,567,555) | 00.42 % (316,474) | 17.32 % (12,907,724) | 17.83 % (13,291,582) |
| Branchpointer |  |  | 1.12 % (836,887) | 00.57 % (423,995) | 00.58 % (428,890) |
| LaBranchoR |  |  |  | 54.5 % (40,621,411) | 28.63 % (21,337,483) |
| RNABPS |  |  |  |  | 41.74 % (31,112,325) |

From the RNA-seq data

| N = 94,806 | SVM-BPfinder | BPP | Branchpointer | LaBranchoR | RNABPS |
| --- | --- | --- | --- | --- | --- |
| SVM-BPfinder | 54.42 % (51,597) | 29.86 % (28,307) | 09.54 % (9,041) | 29.07 % (27,562) | 31.15 % (29,535) |
| BPP |  | 54.83 % (51,985) | 09.93 % (9,416) | 29.38 % (27,852) | 28.96 % (27,456) |
| Branchpointer |  |  | 17.8 % (16,877) | 09.62 % (9,122) | 09.32 % (8,836) |
| LaBranchoR |  |  |  | 54.83 % (51,985) | 34.57 % (32,777) |
| RNABPS |  |  |  |  | 53,92 % (51,124) |

# **Figure S5:** The overlap of natural 3’ ss (True Calls) and controls AG (False Calls) from Ensembl data.


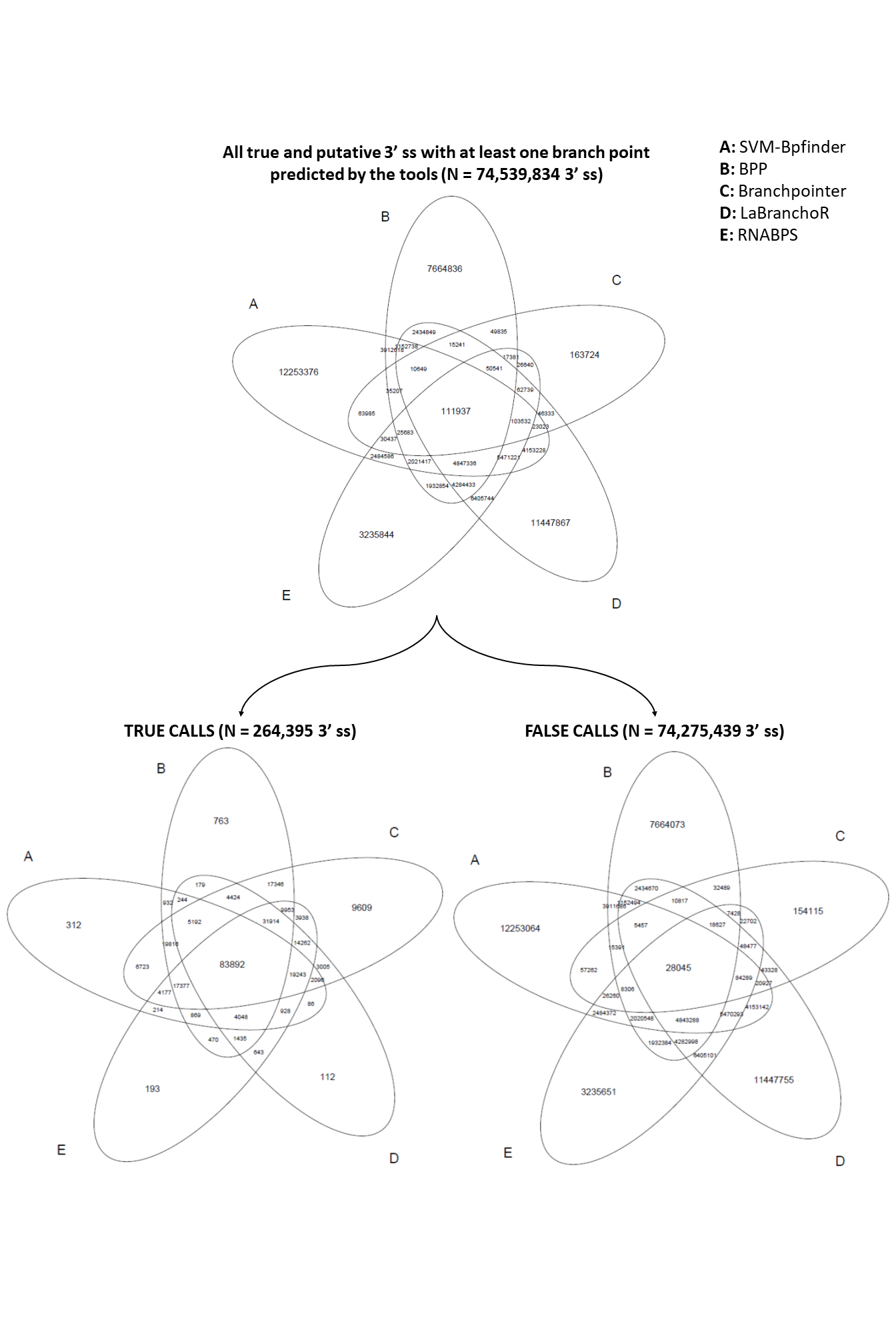


**Figure S5 (continuing):**

| **Score combinaison** | **True calls** | **False calls** | **ratio  True calls/all calls** |
| --- | --- | --- | --- |
| SVM-BPfinder; BPP; Branchpointer; LaBranchoR; RNABPS | 83892 | 28045 | 74.95% |
| SVM-BPfinder; BPP; Branchpointer; RNABPS | 17377 | 8306 | 67.66% |
| BPP; Branchpointer; LaBranchoR; RNABPS | 31914 | 18627 | 63.14% |
| BPP; Branchpointer; RNABPS | 9953 | 7428 | 57.26% |
| SVM-BPfinder; BPP; Branchpointer | 19816 | 15391 | 56.28% |
| SVM-BPfinder; BPP; Branchpointer; LaBranchoR | 5192 | 5457 | 48.76% |
| BPP; Branchpointer | 17346 | 32489 | 34.81% |
| BPP; Branchpointer; LaBranchoR | 4424 | 10817 | 29.03% |
| Branchpointer; LaBranchoR; RNABPS | 14262 | 48477 | 22.73% |
| SVM-BPfinder; Branchpointer; LaBranchoR; RNABPS | 19243 | 84289 | 18.59% |
| Branchpointer; RNABPS | 3938 | 22702 | 14.78% |
| SVM-BPfinder; Branchpointer; RNABPS | 4177 | 26260 | 13.72% |
| SVM-BPfinder; Branchpointer | 6723 | 57262 | 10.51% |
| SVM-BPfinder; Branchpointer; LaBranchoR | 2096 | 20927 | 9.10% |
| Branchpointer; LaBranchoR | 3005 | 43328 | 6.49% |
| Branchpointer | 9609 | 154115 | 5.87% |
| SVM-BPfinder; BPP; LaBranchoR; RNABPS | 4048 | 4843288 | 0.08% |
| SVM-BPfinder; BPP; RNABPS | 869 | 2020548 | 0.04% |
| BPP; LaBranchoR; RNABPS | 1435 | 4282998 | 0.03% |
| BPP; RNABPS | 470 | 1932384 | 0.02% |
| SVM-BPfinder; BPP | 932 | 3911686 | 0.02% |
| SVM-BPfinder; BPP; LaBranchoR | 244 | 1152494 | 0.02% |
| SVM-BPfinder; LaBranchoR; RNABPS | 928 | 5470293 | 0.02% |
| LaBranchoR; RNABPS | 643 | 6405101 | 0.01% |
| BPP | 763 | 7664073 | 0.01% |
| SVM-BPfinder; RNABPS | 214 | 2484372 | 0.01% |
| BPP; LaBranchoR | 179 | 2434670 | 0.01% |
| RNABPS | 193 | 3235651 | 0.01% |
| SVM-BPfinder | 312 | 12253064 | 0.00% |
| SVM-BPfinder; LaBranchoR | 86 | 4153142 | 0.00% |
| LaBranchoR | 112 | 11447755 | 0.00% |

# **Figure S6:** Splicing junctions filtered out from RNA-seq data. Alt 3’ss: alternative acceptor splice sites.


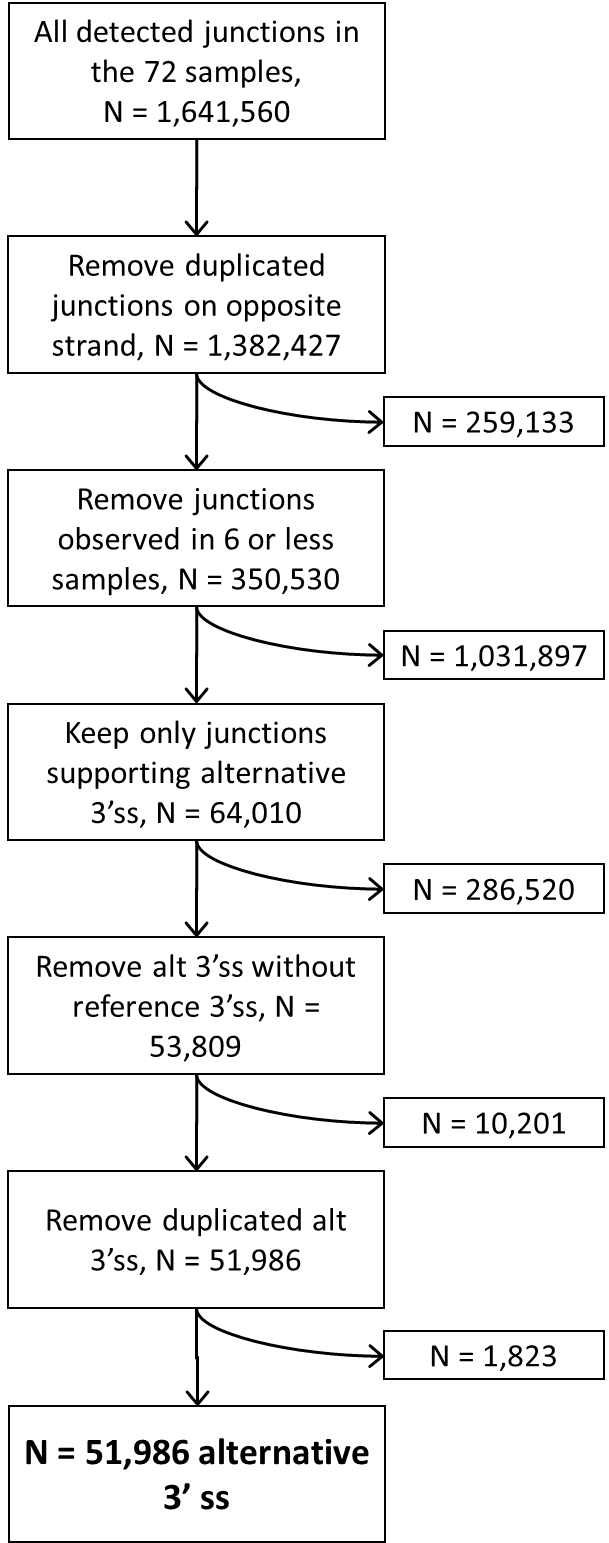


# **Figure S7:** The distribution of the relative expression of alternative 3’ss.

# **Figure S8:** The overlap of alternative 3’ ss (True Calls) and controls AG (False Calls) from our RNAseq data.


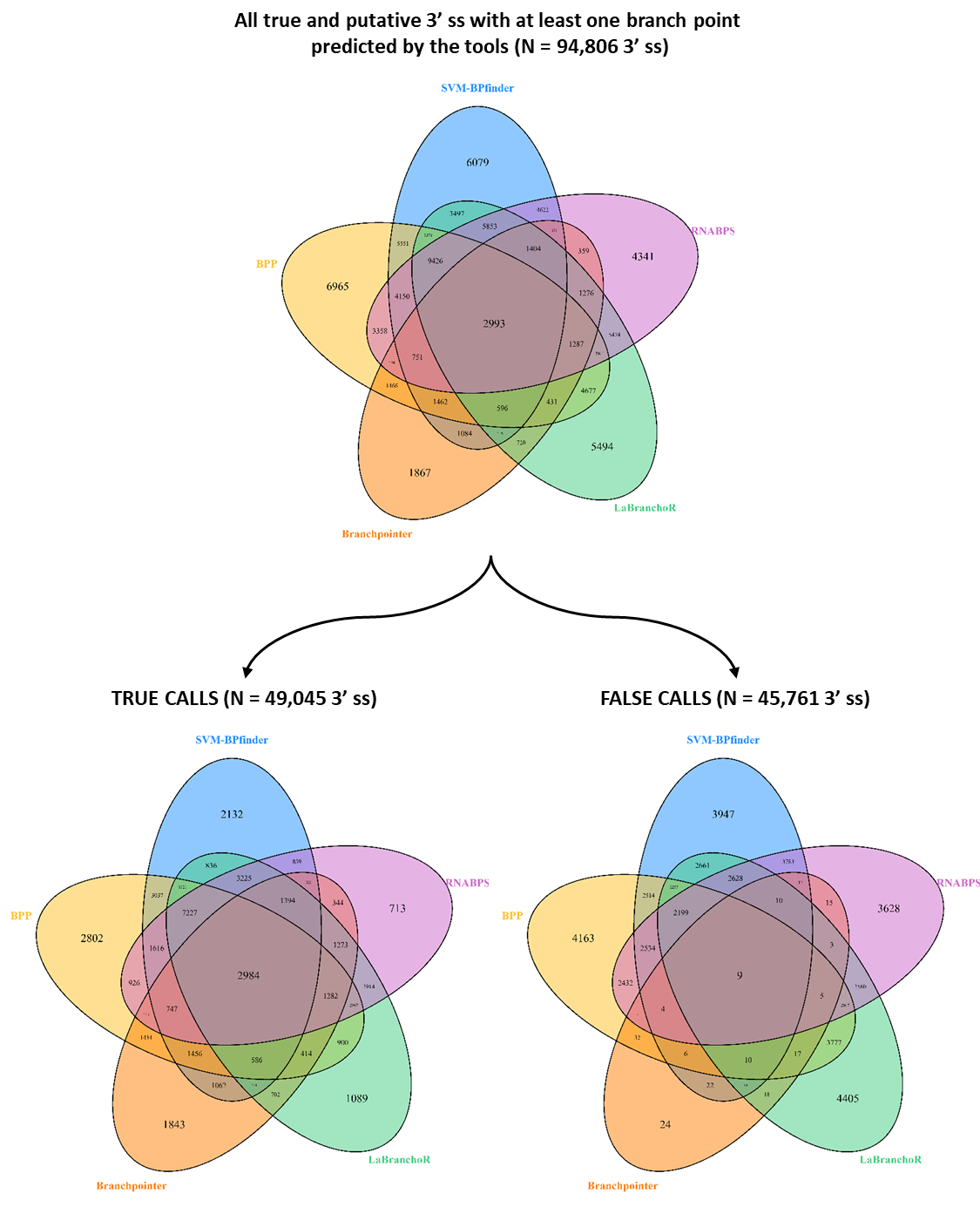


**Figure S8 (continuing):**

| **Score combinaison** | **True calls** | **False calls** | **Ratio  True calls/all calls** |
| --- | --- | --- | --- |
| Branchpointer; LaBranchoR; RNABPS | 1273 | 3 | 99,76% |
| SVM-BPfinder; BPP; Branchpointer; LaBranchoR; RNABPS | 2984 | 9 | 99,70% |
| BPP; Branchpointer; LaBranchoR; RNABPS | 1282 | 5 | 99,61% |
| SVM-BPfinder; BPP; Branchpointer | 1456 | 6 | 99,59% |
| SVM-BPfinder; BPP; Branchpointer; RNABPS | 747 | 4 | 99,47% |
| SVM-BPfinder; Branchpointer; LaBranchoR; RNABPS | 1394 | 10 | 99,29% |
| Branchpointer | 1843 | 24 | 98,71% |
| BPP; Branchpointer; RNABPS | 423 | 7 | 98,37% |
| SVM-BPfinder; BPP; Branchpointer; LaBranchoR | 586 | 10 | 98,32% |
| SVM-BPfinder; Branchpointer | 1062 | 22 | 97,97% |
| BPP; Branchpointer | 1434 | 32 | 97,82% |
| Branchpointer; LaBranchoR | 702 | 18 | 97,50% |
| SVM-BPfinder; Branchpointer; RNABPS | 326 | 10 | 97,02% |
| SVM-BPfinder; Branchpointer; LaBranchoR | 401 | 14 | 96,63% |
| BPP; Branchpointer; LaBranchoR | 414 | 17 | 96,06% |
| Branchpointer; RNABPS | 344 | 15 | 95,82% |
| SVM-BPfinder; BPP; LaBranchoR; RNABPS | 7227 | 2199 | 76,67% |
| BPP; LaBranchoR; RNABPS | 2997 | 2067 | 59,18% |
| SVM-BPfinder; LaBranchoR; RNABPS | 3225 | 2628 | 55,10% |
| SVM-BPfinder; BPP | 3037 | 2514 | 54,71% |
| LaBranchoR; RNABPS | 2914 | 2560 | 53,23% |
| BPP | 2802 | 4163 | 40,23% |
| SVM-BPfinder; BPP; RNABPS | 1616 | 2534 | 38,94% |
| SVM-BPfinder | 2132 | 3947 | 35,07% |
| SVM-BPfinder; BPP; LaBranchoR | 1121 | 2257 | 33,19% |
| BPP; RNABPS | 926 | 2432 | 27,58% |
| SVM-BPfinder; LaBranchoR | 836 | 2661 | 23,91% |
| LaBranchoR | 1089 | 4405 | 19,82% |
| BPP; LaBranchoR | 900 | 3777 | 19,24% |
| SVM-BPfinder; RNABPS | 839 | 3783 | 18,15% |
| RNABPS | 713 | 3628 | 16,42% |

# **Figure S9:** Correlation between the scores (SVM-BPfinder, BPP, Branchpointer, LaBranchoR, RNABPS) and the expression of alternative 3’ss. **A**: SVM-BPfinder (R² = 0.0013, p-value = 1.70x10-16), **B**: BPP (R² = 0.006, p-value = 1.45x10-69), **C**: Branchpointer (R² = 0.0001, p-value = 0.24), **D**: LaBranchoR (R² = 0.0028, p-value = 1.08x10-33), **E**: RNABPS (R² = 0.0062, p-value = 4.14x10-70).

**C**

**B**

**A**

**E**

**D**

# **Figure S10:** Repartition of variants (n = 120) according their position relative to the predicted branch point. Position 0 corresponds to the branch point adenosine. Shown in black are variants affecting splicing, and in grey the variants without effect on mRNA splicing.

# **Figure S11:** Determination of optimal motif (YTRAYNN) length to predict splicing alteration, n = 120 variants. ACC: Accuracy, Pos: relative position in branch point motif, Se: Sensitivity, Sp: Specificity.


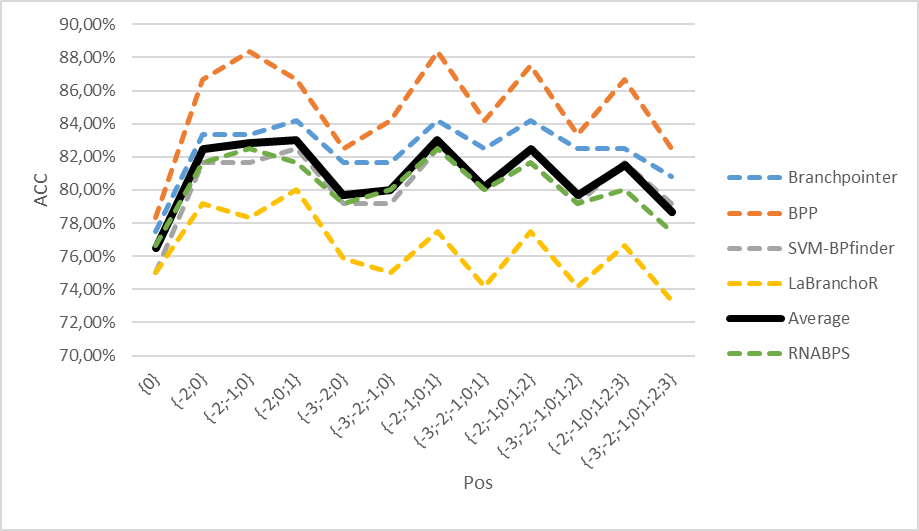


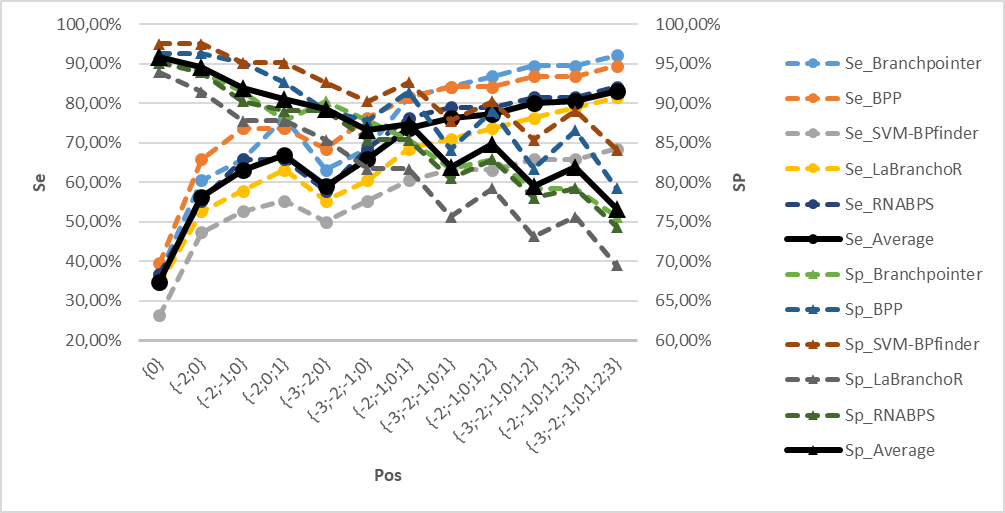


| **Relative position in the motif** | **Sequence motif (7-mer: YTRAYNN)** |
| --- | --- |
| {0} | A |
| {-2;0} | T-A |
| {-2;-1;0} | TRA |
| {-2;0;1} | T-AY |
| {-3;-2;0} | YT-A |
| {-3;-2;-1;0} | YTRA |
| {-2;-1;0;1} | TRAY |
| {-3;-2;-1;0;1} | YTRAY |
| {-2;-1;0;1;2} | TRAYN |
| {-3;-2;-1;0;1;2} | YTRAYN |
| {-2;-1;0;1;2;3} | TRAYNN |
| {-3;-2;-1;0;1;2;3} | YTRAYNN |

# **Figure S12:** Cross-validation (1,000 times) to select the optimal model to predict branch point alteration.

| Acronym of variables | Explanation |
| --- | --- |
| Delta | Variation score between wild Type-mutated |
| MutInPBarea | Is variant located in the 4-mer of BP-predicted |

| Acronym of scores | Complete score names |
| --- | --- |
| HSF | Human Splicing Finder |
| SVM | SVM-BPfinder |
| BPP | Branch Point Predictor |
| Branch | Branchpointer |
| LB | LaBranchoR |
| RNABPS | RNA Branch Point Selection |

**Figure S12 (continuation)**: Likelihood ratio test (LRT) between univariate model and tested model. The univariate model was position of predicted-BP by BPP alone. The red line represents the p-value of 1 %.

# **Figure S13:** Cross-validation (1,000 times) to select the optimal model to predict branch point alteration without the positions of predicted BP for all tools except BPP.

| Acronym of variables | Explanation |
| --- | --- |
| Delta | Variation score between wild Type-mutated |
| MutInPBarea | Is variant located in the 4-mer of BP-predicted |

| Acronym of scores | Complete score names |
| --- | --- |
| HSF | Human Splicing Finder |
| SVM | SVM-BPfinder |
| BPP | Branch Point Predictor |
| Branch | Branchpointer |
| LB | LaBranchoR |
| RNABPS | RNA Branch Point Selection |
